# RNA Structure—A Neglected Puppet Master for the Evolution of Virus and Host Immunity

**DOI:** 10.3389/fimmu.2018.02097

**Published:** 2018-09-19

**Authors:** Redmond P. Smyth, Matteo Negroni, Andrew M. Lever, Johnson Mak, Julia C. Kenyon

**Affiliations:** ^1^Helmholtz Institute for RNA-based Infection Research, Würzburg, Germany; ^2^Faculty of Medicine, University of Würzburg, Würzburg, Germany; ^3^Université de Strasbourg, CNRS, Architecture et Réactivité de l'ARN, UPR9002, F-67000, Strasbourg, France; ^4^Department of Medicine, University of Cambridge, Addenbrooke's Hospital, Cambridge, United Kingdom; ^5^Department of Medicine, Yong Loo Lin School of Medicine, National University of Singapore, Singapore, Singapore; ^6^Institute for Glycomics, Griffith University, Gold Coast, QLD, Australia; ^7^Department of Microbiology and Immunology, Yong Loo Lin School of Medicine, National University of Singapore, Singapore, Singapore; ^8^Homerton College, Cambridge, United Kingdom

**Keywords:** RNA structure, viral evolution, secondary structure, immune evasion, viral RNA

## Abstract

The central dogma of molecular biology describes the flow of genetic information from DNA to protein via an RNA intermediate. For many years, RNA has been considered simply as a messenger relaying information between DNA and proteins. Recent advances in next generation sequencing technology, bioinformatics, and non-coding RNA biology have highlighted the many important roles of RNA in virtually every biological process. Our understanding of RNA biology has been further enriched by a number of significant advances in probing RNA structures. It is now appreciated that many cellular and viral biological processes are highly dependent on specific RNA structures and/or sequences, and such reliance will undoubtedly impact on the evolution of both hosts and viruses. As a contribution to this special issue on host immunity and virus evolution, it is timely to consider how RNA sequences and structures could directly influence the co-evolution between hosts and viruses. In this manuscript, we begin by stating some of the basic principles of RNA structures, followed by describing some of the critical RNA structures in both viruses and hosts. More importantly, we highlight a number of available new tools to predict and to evaluate novel RNA structures, pointing out some of the limitations readers should be aware of in their own analyses.

## Introduction

Mutation rates of viral genomes are extremely high when compared with those of eukaryotic cells; RNA virus polymerases typically possess error rates of 10^−4^ to 10^−6^ per base (1). Such rapid mutation is a strategy by which they can evade host adaptive immune responses (2). Antiviral defenses of the innate immune system, which is less genetically flexible than the adaptive response, enable a very broad range of recognition from which it is difficult for viruses to escape, even given their high error rate. To counter the innate immune system, viruses have developed strategies to block its activation. This arms race prompts the immune system to develop counter measures to recognize and eliminate the virus, whilst viruses that survive and transmit successfully are those that have evolved to escape it. When considering the evolutionary pressures on both virus and host, research has often focused on the protein sequences needed by each. A recent explosion in our understanding of the functions of RNA, however, leads us to consider instead the role of RNA itself in driving the evolution of viruses and of human immunity.

**Figure 1:**
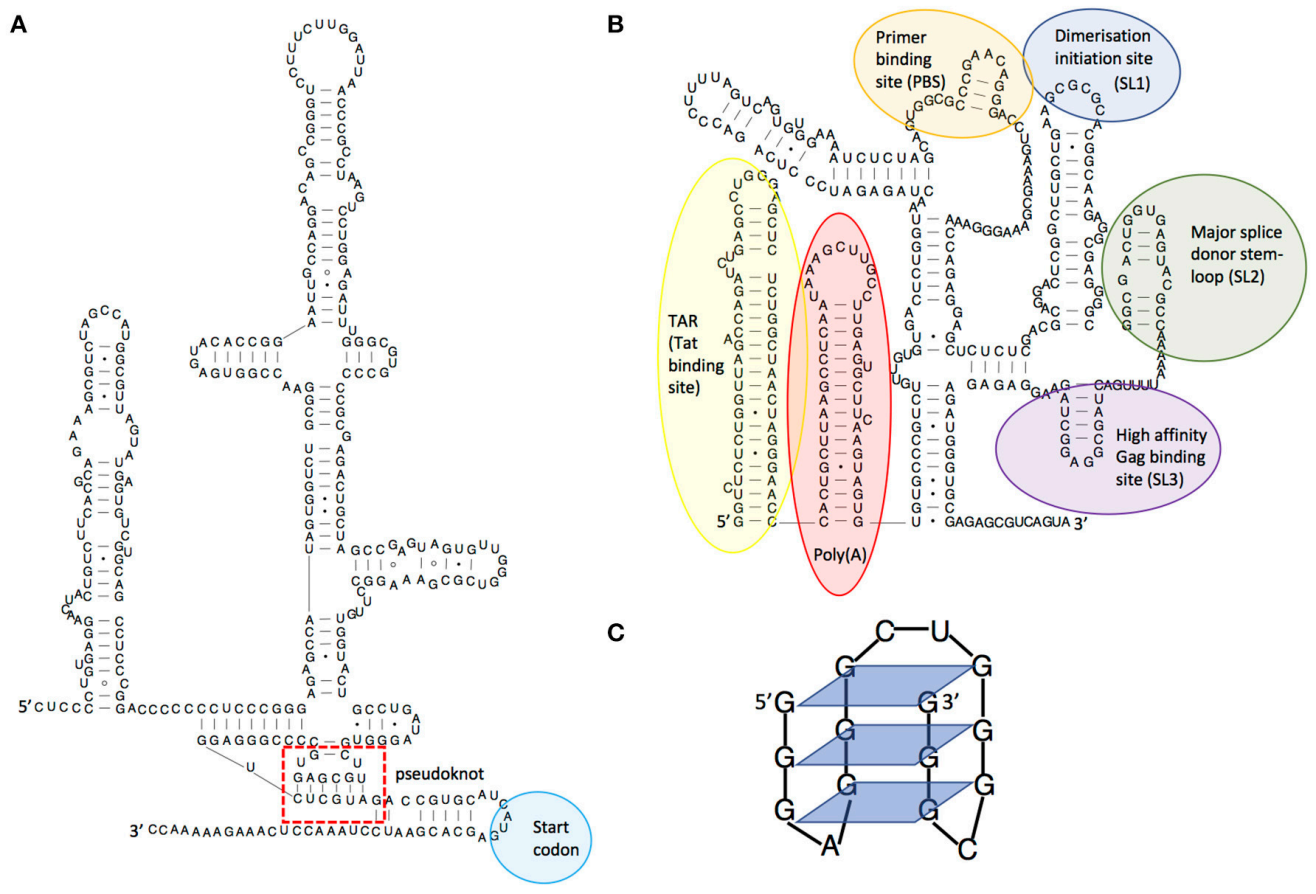
Examples of RNA structures. **(A)** Structural diagram of the hepatitis C virus IRES structure, showing its pseudoknot. Adapted from Malygin et al.(4), **(B)** Structural model of the HIV-1 5'leader RNA, highlighting some of the important RNA structures it contains. Adapted from Kenyon et al. (5), **(C)** An RNA guanine quadruplex structure, showing the 4-way bonds between guanines in each plane. Adapted from Frees et al. (6).

RNA is a truly multifunctional molecule. It directs ribosomes (themselves RNA based enzymes) to produce proteins, but also regulates cellular activity by interacting directly with proteins or nucleic acids and by catalyzing biochemical reactions (ribozymes). Indeed, RNA is now implicated in almost every cellular process, including immune defense. It is also recognized as being key to viral infection processes. RNA multifunctionality comes from its ability to fold into complex three-dimensional structures that can often switch conformation to effect different functions such as binding other RNA molecules or proteins. The initial fold of an RNA molecule depends primarily on its sequence and is established by Watson-Crick pairing of complementary bases into stem-loop structures that then orientate themselves relative to one another [for a general review of RNA structure see (3)]. This three-dimensional positioning can be stabilized by non-canonical interactions or structures such as pseudoknots, which occur where the nucleotides of a loop region base pair intramolecularly with complementary nucleotides. An example is shown in Figure 1A, within the complex structural element known as an IRES (internal ribosome entry site). As for proteins, single nucleotide mutations can alter the three-dimensional structure of the RNA, with corresponding deleterious or positive effects on its function; RNA structures are hence substrates for, and drivers of, viral evolution. For example, random mutation may confer a new beneficial function on a given structure that is then selectively favored by evolution.

Viruses are known to be extremely thrifty with their genomes to maximize replication speed, using strategies such as overlapping reading frames and polycistronic mRNAs. Similarly, they often contain functional RNA structures within both coding and non-coding regions. For example, the first 500 nucleotides of the HIV-1 genome is densely packed with structured domains that control key steps of the replication cycle including transcription, translation, export, packaging, and reverse transcription (7–9) and structural switches that aid their regulation (5, 10–12). Functionality encoded within an RNA structure is often a requisite for successful initiation or completion of viral replication. Many viruses, particularly those of the *picornaviridae*, initiate translation from IRES elements (13). RNA structures facilitating frameshifting enable viruses to encode multiple proteins from a single RNA (14). Additionally, viral RNA structures may directly or indirectly impact cellular immunity. In HIV-1, the transactivation response element (TAR, Figure 1B) regulates transcription of the genomic RNA and gene expression (15) thereby playing a central role in determining the level of virus detected by the immune system; at its most extreme leading to complete evasion of the immune response through latency (16). Frameshift structures or splicing regulators qualitatively and quantitatively manage the amounts of proteins produced by viruses, and hence those that are seen by the immune system (17). For example, some strains of Influenza A virus encode a second open reading frame (ORF) in segment three which is accessed by a low frequency +1 ribosome frameshifting event (18). This ORF produces PA-X, a protein which modulates inflammatory, apoptotic, and T-lymphocyte signaling pathways (18). Viruses with larger genomes can even produce their own microRNAs (19) or long non-coding RNAs (lncRNAs) that control cellular functions (20, 21). Dengue virus expresses a subgenomic RNA that has been shown to inhibit interferon expression by binding to TRIM25 (22). This subgenomic RNA is produced when a cellular 5′-to-3′ exoribonuclease stalls at a stable pseudoknot RNA structure in the 3′UTR; small substitutions within this structure modulate viral fitness and pathogenicity through their effects on the immune system. The dengue 3'UTR also folds differently in humans and insects (23) leading to production of different immunomodulatory non-coding RNAs in each host type (22, 23). This is one mechanism by which the same viral genome can both effectively replicate in human and insect cells and counteract these two divergent immune systems.

The immune system senses viral RNA using different mechanisms, including the recognition of viral single-stranded RNA by Toll-like receptors (24). The importance of RNA structures in viral replication is so fundamental that they are directly recognized by the innate immune response. Not only has the innate immune system evolved to recognize double-stranded RNA (dsRNA) within viral genomes or genomic replication intermediates, often via MDA5, it has also evolved to recognize the double-stranded parts of conserved RNA elements like IRESs (13) or 3' stem-loops, via RIG-I [reviewed in (25)]. The importance of this in maintaining broad antiviral defense is reflected in the fact that such RNA structures are also formed by DNA viruses in their protein-coding RNAs. The cellular double-stranded RNA recognition system not only leads to production of interferon, but was recently shown to upregulate NKG2D ligand, thus alerting NK cells to the presence of virus and enabling destruction of the infected cell (26). Viruses have evolved strategies to mask recognition of their RNAs, such as 5' cap-snatching (27), but their need to maintain certain critical RNA structures means that they struggle to avoid recognition entirely.

## Viral evolution to evade an immune response may be constrained or facilitated by RNA structure

The presence of essential conserved viral RNA structures can constrain the ability of viruses to evolve and to evade the immune response. Some RNA viruses have optimized their genome structure and organization to facilitate viral evolution during co-infection of the same cell with different viral strains. One widespread strategy is genome segmentation leading to reassortment (seen in rotaviruses and influenza viruses) (28). Another common strategy is template switching during replication leading to recombination and the formation of genome chimeras (seen in retroviruses) (29). Reassortment and recombination are non-random processes that are known to depend on RNA sequence and structure, but the underlying mechanisms are often poorly understood. Both processes may be facilitated by inter-molecular interactions (30, 31), and evolution can be both promoted or inhibited by intra-molecular RNA structure.

In HIV-1, each virion contains two copies of the RNA genome, which in the case of co-infection of the same cell can originate from two different proviruses. These are non-covalently joined as a dimer via stem-loop 1 (SL1, Figure 1B). During viral replication, reverse transcriptase (RT) switches template, thereby adding template switching generated errors to its inherently low fidelity, producing the genetic diversity that allows HIV-1 to rapidly escape the immune system and antiretroviral therapy (2). Sequence incompatibility preventing formation of heterodimers of genomic RNA at SL1 has been shown to be a major restriction to inter-subtype recombination, with only a 3-nt sequence difference being sufficient to disrupt this (32). When considering distantly-related strains of HIV with compatible SL1 sequences, the main factor governing recombination is the degree of local sequence similarity (33, 34) However, in more closely-related viral sequences, RNA structures strongly influence recombination locally (35–37). This has been shown to be the case for well-defined RNA structures within *env* such as the C2 hairpin and the Rev responsive element (RRE) (38). It has been suggested that this evolved to favor the stepwise folding of proteins during translation, but it has also been shown to favor the occurrence of recombination in these same regions (37). As a consequence, recombination may shuffle whole domains of proteins thus generating structural variants that escape immune recognition, particularly for quaternary epitopes generated by the juxtaposition of different protein domains. RNA structures and sequences have also been shown to influence the fidelity of reverse transcription such that stable secondary structures enhance the number and type of mutations incorporated (39, 40). The regions of *env* encoding the external parts of the viral surface glycoprotein gp120 are under strong positive selection by the humoral immune system. Perhaps counterintuitively they present a lower degree of RNA structure; however, this can be accounted for by such rapid viral mutation that the RNA is unable to conserve base-pairing. As studies have shown, poorly structured RNA regions are reverse transcribed with higher fidelity, which paradoxically limits the rate of introduction of mutations in these highly variable regions of the genome (39).

RNA viruses with segmented genomes can undergo reassortment, leading to the exchange of entire gene segments, potentially giving rise to new viral strains to which humans have no previous immunity. In influenza A, reassortment has been historically associated with the emergence of pandemic strains, including the most recent H1N1 2009 pandemic which contained influenza gene segments from human, avian and swine lineages (41, 42). It is thought that packaging sequences within each gene segment direct their selective incorporation into virus particles (43, 44) and recent work suggests a mechanism where packaging signals mediate RNA-RNA interactions that would guide their incorporation. It therefore follows that any preferential interactions or incompatibilities between vRNP segments would then regulate genetic reassortment and influenza evolution (45). It is possible that improved understanding of this process would help to better predict the emergence of pandemic influenza.

In addition to genome diversification through recombination and reassortment, RNA structures influence immune escape by modulating the viral proteome. The use of frameshifting or alternative start codons is often controlled by viral RNA structures, resulting in the translation of viral peptides or proteins in a different reading frame. The resulting peptides are often antigenic and may act to dilute the presentation of conventional viral peptides on the surface of the infected cell (46). In some viruses there is an evolutionary constraint to maintain translation-impeding RNA structures in genes that encode good T cell epitopes, thus maintaining their translation at levels too low to trigger T cell recognition and killing. The EBNA-1 RNA from Epstein-Barr virus for example regulates its own translation *in cis* (47) and the evolutionary pressure on this gene comes from the need to maintain G-quadruplex RNA structures (Figure 1C) that act as “steric blocks” to ribosomes. When these structures are destabilized, cells infected with the resulting mutant virus are more readily seen by T cells than those infected with wild-type virus (48).

Viruses also appear to be under pressure to maintain unstructured regions in their genome: there is a bias in HIV-1 toward the use of A's in the retroviral genome; this biases codon usage and ultimately even the amino acid composition of the viral proteins (49). Adenosines are vastly overrepresented in the single-stranded regions of RNA and underrepresented in double-stranded regions; their only binding partner, U, can also pair with G, which may explain the single-stranded nature of A-rich regions. Artificial introduction of extensive synonymous A to G mutations in *pol* led to increased stability of the dimeric genome inside the virion, and reduced reverse transcription as a result (50). The signature distribution of Adenosine frequency and its relation to local RNA structure was thought to be maintained by the influence of RNA secondary structures on reverse transcription. Changing the A ratio in local areas by only including codons found in natural isolates of HIV-1, did not affect replication efficiency *in vitro* (51) however it is possible that *in vivo* viruses use this strategy of maintaining parts of their genome as single-stranded in order to avoid innate immune recognition by RIG-I or MDA5. Many viruses also need to maintain a low number of CpG dinucleotides in their genome in order to avoid recognition by the zinc-finger antiviral protein (ZAP) (52). When HIV-1 codon usage was humanized, affecting the native RNA structure, a reduced IFN-α/β response was observed (53), suggesting that the maintenance of specific structures in the viral genome comes at the cost of greater recognition by the innate immune response. Despite this, the viral genome apparently undergoes positive selection for the maintenance of many specific RNA structures: when synonymous mutations were introduced extensively into the viral genome a decrease in infectivity was observed that could be attributed to an expected alteration of splicing pattern and/or modification of RNA structures (50, 54).

## The roles of viral and cellular RNA structure in the evolution of human immune responses and the human immune system

In terms of the evolution of the human genome, the degree of variation at the MHC class I locus is positively correlated with local pathogen richness, for which viruses are postulated to play an important role. This is particularly evident for HLA B (55). The importance of RNA structure in influencing the generation of T cell epitopes, either through translational enhancing, blocking, or frameshifting mechanisms, means that RNA structures within viruses must have influenced the evolution of the HLA locus. For example, macaques that have the correct MHC-I allele to present an antigenic cryptic peptide derived from the *env* ORF are better able to control simian immunodeficiency virus (SIV) infection (56) which would be expected to be a driver for the maintenance of this MHC allele within the population. As mentioned above, the mechanisms controlling the generation of cryptic translational products are often RNA structure-dependent.

Viral RNA structures have also influenced the evolution of the innate immune system; as previously discussed, hallmarks of viral RNAs are targeted by conserved RNA-binding proteins such as RIG-I. More specific antiviral proteins have also evolved, however. APOBEC3 proteins target retroviral genomes and are incorporated into viral particles (57). These host cell-derived cytidine deaminases bind to the viral RNA and mutate it during the reverse transcription process, leading to non-functional virus. It has been reported that regions of the genome under strong purifying selection present an underrepresentation of APOBEC3 target sequences, a signature of a strong pressure for limiting the occurrence of mutations in certain regions of the genome (39). Retroviruses have also developed direct strategies to counteract these proteins, often by encoding proteins that bind to them directly. Interestingly, APOBEC3s have recently been shown to bind to the same motifs in the viral RNA genome as the viral NC structural protein involved in genome packaging. This suggests a competitive relationship may have developed between host cell and viral factors, for binding to the same viral RNA structures (58).

## Novel ways to explore RNA structure and function

RNA functionality is best understood through its structure, but RNA structure determination is extremely challenging. Although it is formed based on simple base pairing rules, for RNA molecules of biologically relevant sizes there are an astronomical number of possible structural permutations, meaning that RNA structure cannot be predicted easily from base pairing rules alone. Biophysical methods such as crystallography (59) and NMR (60) are each able to determine RNA structure at atomic resolution but both have difficulty with large RNA substrates. This is evidenced by the paucity of atomic resolution RNA structures, compared to their protein equivalents. For example, the RCSB databank holds structural data for over 100,000 proteins, but contains only around 1000 RNA structures. This difficulty arises because RNA molecules tend to adopt long flexible shapes with weak tertiary interactions that are prone to misfolding (61). Furthermore, the negatively charged phosphate groups on the surface of an RNA molecule can impose technical challenges as they hinder crystal packing. Newer techniques are emerging to address this gap including small-angle X-ray scattering (62), single molecule FRET (63, 64), and atomic force microscopy (65).

RNA secondary structure is currently most commonly resolved using a combination of (i) phylogenetic approaches (ii), structure prediction algorithms, and (iii) experimental methods with chemical/enzymatic probes. Whilst these methodologies cannot determine RNA structure at atomic resolution, they are nevertheless able to generate models that provide useful biological insights (8, 66, 67). Indeed, RNA structure determination is currently undergoing a revolution thanks to advances in next generation sequencing technology that have transformed traditional biochemical assays into powerful tools that can characterize thousands of RNA structures in single experiments (68–71). The most widely used methods take advantage of chemical probes, such as dimethyl sulfate (DMS) and selective 2′-hydroxyl acylation analyzed by primer extension (SHAPE) reagents, that differentially react with single stranded vs double stranded RNA. Knowledge of whether a nucleotide is likely to be base paired or not can significantly improve the accuracy of RNA structure predictions from thermodynamic folding algorithms when included as an energetic consideration in the modeling programme, known as a pseudo free energy parameter (72). For example, if chemical probes show a nucleotide is single-stranded, the modeling algorithm adds an energetic penalty to structures that include it in a double-stranded region. The programme then displays the most energetically favorable structures, that fit all of the data best. Several chemical probes can penetrate cells and virions, which is important for understanding RNA function *in vivo*, such as the binding sites of regulatory proteins (9, 73). Further characterization of RNA structure-function relationships can be obtained using specialized approaches, such as mutational interference mapping experiment (MIME) (74) and cross-linking SHAPE (XL-SHAPE), where protein binding sites are mapped using UV cross-linking in parallel with SHAPE probing (75), and by CLIP (crosslinking-immunoprecipitation sequencing) related methodologies (76). More recently, RNA proximity ligation has emerged as a new class of RNA structural probing technique for the direct detection of long-range base pairing or inter-molecular interactions (77–84). These types of interactions are commonly found in viral genomes/regulatory RNAs and are difficult to identify with other methodologies. As the immune system is known to be regulated by non-coding RNAs (85), the ability to detect direct interactions between viral and cellular RNAs will be particularly important for future understanding of virus-host interactions.

## Author contributions

JM conceived the review. RS, MN, AL, JM, and JK wrote the manuscript.

### Conflict of interest statement

The authors declare that the research was conducted in the absence of any commercial or financial relationships that could be construed as a potential conflict of interest.
